# Oxidative Stress, Gut Microbiota, and Extracellular Vesicles: Interconnected Pathways and Therapeutic Potentials

**DOI:** 10.3390/ijms26073148

**Published:** 2025-03-28

**Authors:** Bo Ma, Muttiah Barathan, Min Hwei Ng, Jia Xian Law

**Affiliations:** Department of Tissue Engineering and Regenerative Medicine, Faculty of Medicine, Universiti Kebangsaan Malaysia, Cheras, Kuala Lumpur 56000, Malaysia; p138160@ukm.edu.my (B.M.); barathanmuttiah@ukm.edu.my (M.B.); angela@ppukm.ukm.edu.my (M.H.N.)

**Keywords:** oxidative stress, dysbiosis, gut microbiota, oxidative DNA damage, extracellular vesicle

## Abstract

Oxidative stress (OS) and gut microbiota are crucial factors influencing human health, each playing a significant role in the development and progression of chronic diseases. This review provides a comprehensive analysis of the complex interplay between these two factors, focusing on how an imbalance between reactive oxygen species (ROS) and antioxidants leads to OS, disrupting cellular homeostasis and contributing to a range of conditions, including metabolic disorders, cardiovascular diseases, neurological diseases, and cancer. The gut microbiota, a diverse community of microorganisms residing in the gastrointestinal tract, is essential for regulating immune responses, metabolic pathways, and overall health. Dysbiosis, an imbalance in the gut microbiota composition, is closely associated with chronic inflammation, metabolic dysfunction, and various diseases. This review highlights how the gut microbiota influences and is influenced by OS, complicating the pathophysiology of many conditions. Furthermore, emerging evidence has identified extracellular vesicles (EVs) as critical facilitators of cellular crosstalk between the OS and gut microbiota. EVs also play a crucial role in signaling between the gut microbiota and host tissues, modulating immune responses, inflammation, and metabolic processes. The signaling function of EVs holds promise for the development of targeted therapies aimed at restoring microbial balance and mitigating OS. Personalized therapeutic approaches, including probiotics, antioxidants, and fecal microbiota transplantation-based strategies, can be used to address OS-related diseases and improve health outcomes. Nonetheless, further research is needed to study the molecular mechanisms underlying these interactions and the potential of innovative interventions to offer novel strategies for managing OS-related diseases and enhancing overall human health.

## 1. Introduction

Oxidative stress (OS), defined as an imbalance between reactive oxygen species (ROS) and the body’s antioxidant defenses, plays an important role in the progression of metabolic, inflammatory, and neurodegenerative disorders [1]. Conversely, the gut microbiota, a diverse community of microorganisms in the gastrointestinal tract (GIT), is equally crucial for health, supporting immune regulation, metabolism, and pathogen defense [2,3]. A bidirectional relationship exists between OS and the gut microbiota, in which controlled ROS production can play a role in maintaining gut homeostasis and immune function [3]. A balanced level of ROS helps regulate microbial communities, promoting beneficial microbes that support digestion, immune defense, and the prevention of pathogenic overgrowth, thereby contributing to a healthy gut microbiome, whereas excessive ROS can damage the gut epithelium and disrupt microbial balance, leading to dysbiosis [4]. In turn, an imbalanced microbiota will further exacerbate OS by promoting inflammation or reducing microbial-derived antioxidants, such as short-chain fatty acids (SCFAs) [5]. This cycle contributes to disease progression, underscoring the importance of strategies to restore redox balance and gut microbiota homeostasis. Additionally, extracellular vesicles (EVs), particularly microbial-released EVs are emerging as pivotal mediators in the intricate relationship between OS and gut microbiota [6]. These nanosized vesicles serve as carriers of diverse bioactive molecules, including proteins, lipids, nucleic acids, and metabolites, enabling intercellular communication and extending the functional influence of the gut microbiota beyond the GIT [7]. Recent studies have highlighted the interconnectedness between OS and gut microbiota dysbiosis, demonstrating how these factors can influence disease progression through inflammatory pathways [8]. Additionally, EVs have emerged as potential mediators of intercellular communication in response to OS. Research has shown that microbial-derived EVs play a fundamental role in regulating redox balance in the gut and may either protect against or exacerbate oxidative damage, depending on the context of the disease [7].

**Figure 1:**
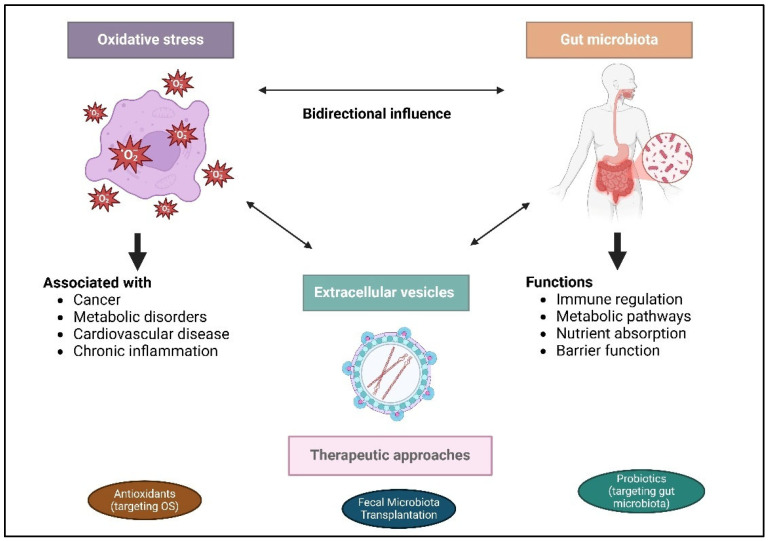
Graphical abstract depicting the bidirectional interplay between OS and gut microbiota in health and disease, with EVs serving as mediators.

This review aims to synthesize existing knowledge on the interactions between OS, gut microbiota, and EVs, exploring how these relationships influence disease progression. Additionally, this review seeks to identify potential therapeutic approaches that leverage these interactions, including probiotics, prebiotics, antioxidants, EV-based therapies, and microbiota-targeted strategies, to alleviate diseases and improve health outcomes. The central hypothesis of this review is that OS, the gut microbiota, and EVs are interconnected. Understanding the mechanisms underlying this interaction, particularly the role of EVs in mediating communication between OS and the microbiota, could lead to the development of targeted interventions aimed at restoring microbial balance and reducing OS. These insights offer novel therapeutic strategies for managing OS-related disease. This review will integrate existing findings, highlight gaps in current knowledge, and propose future research directions, particularly focusing on the potential of EVs to bridge the gap between gut microbiota and OS, with promising clinical implications. Figure 1 is a graphical abstract that illustrates the bidirectional interplay between OS and the gut microbiota in health and disease. The left side shows ROS linked to metabolic disorders, cardiovascular diseases, cancer, and inflammation. The right panel depicts the roles of the gut microbiota in immunity, metabolism, nutrient absorption, and barrier function. Centrally, EVs mediate crosstalk, influencing disease pathogenesis. The bottom highlights therapeutic strategies such as antioxidants, probiotics, and fecal microbiota transplantation to counter OS and restore microbial balance for improved health.

## 2. Materials and Methods

A literature search was conducted using PubMed, Scopus, and Google Scholar to identify relevant articles on OS, gut microbiota, and EVs. Keywords such as “oxidative stress”, “microbial-derived extracellular vesicles”, “dysbiosis”, and “gut microbiota” were used in various combinations. The search was restricted to peer-reviewed articles published in English between 2010 and 2023. A total of approximately 250 articles were initially identified. After screening for relevance, around 80 studies were included in the final review. The inclusion criteria focused on studies examining the interplay between OS EVs and microbial metabolites, research articles published in peer-reviewed journals, and studies focusing on the gut microbiota and its role in OS. The exclusion criteria included articles unrelated to the main topic, review papers, non-peer-reviewed studies (preprints, conference abstracts), and studies published in languages other than English. The selection process involved an initial screening of titles and abstracts, followed by a detailed review of the full texts for relevance. Potential biases and limitations, such as the exclusion of non-English articles, were acknowledged.

## 3. Oxidative Stress (OS)

An imbalance between ROS and antioxidants is an essential factor in the development of OS. ROS are highly reactive molecules derived from oxygen, including superoxide anions (O_2_^−^), hydrogen peroxide (H_2_O_2_), and hydroxyl radicals (•OH) [9]. Their overproduction can occur when physiological processes—such as mitochondrial respiration, inflammatory responses, or environmental factors like radiation and toxins—exceed the capacity of the antioxidant defense system to neutralize them [9,10].

The body’s antioxidant defenses, including enzymatic systems such as superoxide dismutase (SOD), catalase (CAT), and glutathione peroxidase (GPx), work to neutralize ROS and prevent oxidative damage [11]. However, when ROS levels exceed the capacity of these defenses, OS occurs, leading to lipid peroxidation, protein oxidation, and DNA fragmentation. These processes compromise cellular integrity and are implicated in the pathogenesis of various diseases [12].

The intricate interplay between ROS and antioxidants highlights the critical need to maintain a delicate balance in cellular homeostasis. Therapeutic strategies, such as antioxidant supplementation and lifestyle modifications, may help restore this balance, potentially mitigating ROS-related damage and enhancing metabolic stability. However, further research is needed to explore targeted interventions and assess their long-term effects on the oxidative stress-related diseases.

## 4. Gut Microbiota: Structure and Function

The human gut microbiota is a highly complex and dynamic community composed of more than 100 trillion microorganisms, including bacteria, archaea, fungi, viruses, and small eukaryotes [13]. The key bacterial phyla in the human gut microbiota include *Bacillota* (Firmicutes), *Bacteroidota* (Bacteroidetes), *Actinomycetota* (Actinobacteria), *Pseudomonadota* (Proteobacteria), *Fusobacteriota* (Fusobacteria), and *Verrucomicrobiota* (Verrucomicrobia) [14]. These phyla play crucial roles in various physiological processes, such as digesting dietary fibers, producing SCFAs, maintaining gut barrier integrity, modulating immune responses, regulating lipid metabolism, preserving energy homeostasis, enhancing host metabolism, and suppressing pathogenic microorganisms [15,16]. Other components of the gut microbiota, including archaea, fungi, and viruses, also contribute to this intricate ecosystem [17]. For example, archaea such as *Methanobrevibacter smithii* play a vital role in energy extraction by participating in metabolic processes like hydrogen utilization [18]. Fungi, such as *Candida* and *Saccharomyces*, are a smaller yet important component of the gut ecosystem, involved in immune regulation and carbohydrate digestion [19]. The gut virome is a diverse and dynamic community of eukaryotic and prokaryotic viruses, with prokaryotic viruses (phages) accounting for over 90% of its composition, primarily dominated by DNA viruses like Caudovirales and Microviridae [20]. RNA phages, while less abundant in the gut virome, display significant diversity, indicating their ecological importance. Among DNA phages, CrAssphages from the Intestiviridae family and Crassvirales order dominate the gut virome, contributing to 22–90% of its composition [21]. However, despite their prevalence, the specific health implications of CrAssphages remain largely unclear, warranting further investigation into their potential roles in gut microbiota dynamics and host health [20]. In addition, eukaryotic viruses, which comprise less than 10% of the gut virome, include latent DNA viruses (such as herpesviruses, anelloviruses, and adenoviruses) and rare RNA viruses (including plant viruses, coronaviruses, sapoviruses, and rotaviruses), which are often linked to infections, regulate bacterial populations, and influence microbial diversity [22]. Eukaryotic viruses also maintain gut homeostasis by activating pathways like TLR3/TLR7 and RIG-I, promoting anti-inflammatory responses and epithelial repair [23]. The virome evolves with age, starting with high phage diversity and low bacterial diversity at birth and transitioning to a stable, individualized composition in adulthood [24]. The gut virome varies with geography, diet, and lifestyle and plays a critical role in health, development, and disease pathogenesis [25]. Conversely, eukaryotic organisms like Blastocystis also inhabit the gut, although their role in health and disease is still being explored [26].

Studies across various populations, including a cross-sectional study of Japanese individuals ranging from one year to more than 100 years, have identified distinct microbiome profiles at different life stages [27]. For example, infants exhibit a characteristic microbiome prior to weaning, which transitions to a more diverse profile with the introduction of solid foods. This diversity continues to develop until early adulthood, stabilizing before showing a decline in richness, particularly pronounced in those more than 80 years, after peaking around 65 years [28]. Similar findings from a Swedish cohort corroborate these observations, highlighting unique microbiome characteristics in young children that resemble the oral microbiota and a transition to a more adult-like microbiome in adolescents [29]. Individual uniqueness increases at the extremes of age, likely reflecting adaptations to diet and environment. Remarkably, individuals more than 100 years old display distinct gut microbiome profiles characterized by greater diversity and higher abundance of health-associated taxa, such as *Christensenellaceae* and *Akkermansia* [30]. Microbial composition varies along the GIT, with a higher density in the colon due to fiber fermentation. Factors like diet, age, health status, antibiotic use, and genetics influence the composition of the gut microbiota [31]. A fiber-rich diet supports beneficial bacteria, while high-fat or processed foods can lead to dysbiosis, promoting pro-inflammatory species [32]. Host genetics significantly influence the gut microbiome composition by regulating metabolism, immune responses, and the removal of unwanted microbes, fostering a balanced microbial community. Bovine studies have shown that host genetics, particularly the paternal genome, exert a strong impact on the preweaning calf gut microbiota. Sire (father) breed composition had a greater impact than dam (mother) breed composition on the structure of the microbiota and immune parameters, such as plasma IgG1 levels. Brahman-sired calves had reduced numbers of pathogenic bacteria and increased numbers of butyrate-producing bacteria, which are beneficial for gut health. Single nucleotide polymorphisms (SNPs) in mucin genes (MUC4, MUC12, MUC13, and MUC20) are correlated with microbiota composition, indicating a genetic basis for gut barrier protection [33]. Notably, variations in mucin-encoding genes are associated with breed composition and the prevalence of mucin-degrading bacteria in the gut. Antibiotic use can lead to reduced microbial diversity, altered metabolic activity, and the selection of antibiotic-resistant organisms [34]. These changes can result in immediate effects, such as antibiotic-associated diarrhea and *Clostridioides difficile* infections, as well as long-term consequences for immunity, metabolism, and overall health [35]. Table 1 provides a concise overview of how gut microbiota dysbiosis is linked to different health conditions, including gastrointestinal disorders, mental health issues, and cardiovascular diseases.

## 5. Interactions Between OS and Gut Microbiota

OS and gut microbiota are closely associated and influence the progression and development of numerous diseases [49]. The trillions of microbes that constitute the gut microbiota play a role in maintaining redox balance through the regulation of the immune response, nutrient metabolism, and the production of antioxidants [4]. However, an imbalance in the gut microbiota (dysbiosis) can lead to overproduction of ROS, weakening of antioxidant defenses, and inflammation, DNA damage, and epigenetic alterations linked to metabolic diseases [50]. This disruption jeopardizes cellular homeostasis and triggers inflammatory responses, further fueling disease progression. Dysbiosis also disrupts these essential functions, leading to a cascade of effects that may compromise cellular homeostasis and provoke inflammatory responses [51]. These responses can occur through direct interactions between microbial cells and host tissues or indirectly via bacterial metabolites such as SCFAs, lipopolysaccharides (LPS), and bile acids [52]. This interplay is particularly evident in intestinal diseases, where dysregulated gut microbiota increase ROS levels, influencing conditions like colorectal cancer (CRC) and inflammatory bowel diseases (IBD) [53].

Gut microbiome dysbiosis, marked by a loss of microbial diversity and an increase in pro-inflammatory species, significantly disrupts the delicate balance of metabolites that are crucial for maintaining cardiovascular health [54]. In a healthy gut, metabolites such as SCFAs, including acetate, propionate, and butyrate, play a pivotal role in reducing inflammation, regulating blood pressure, and maintaining the integrity of the intestinal barrier [55]. Dysbiosis leads to a decline in SCFA levels, compromising the gut barrier and allowing bacterial toxins and inflammatory products to enter the systemic circulation, triggering widespread inflammatory responses [56]. Additionally, dysbiosis affects the metabolism of tryptophan, an essential amino acid, leading to the production of harmful metabolites like indoxyl sulfate, which promotes vascular inflammation, while reducing beneficial compounds like indole-3-propionic acid, which protects against oxidative damage [57]. Furthermore, metabolites like trimethylamine N-oxide (TMAO), derived from dietary choline, L-carnitine, and betaine, are elevated during dysbiosis, exacerbating platelet activation, cholesterol transport, and atherosclerotic plaque formation [58]. LPS further intensifies systemic inflammation by inducing cytokines and ROS, damaging vascular endothelial cells, and accelerating atherosclerosis [59]. The reduction of secondary bile acids during dysbiosis disrupts lipid metabolism and anti-inflammatory signaling via TGR5 and FXR receptors, while decreased nitric oxide (NO) production enhances OS and nitro-oxidative damage, promoting hypertension and cardiac infarctions [60]. These interconnected pathways highlight how gut dysbiosis fosters systemic inflammation, endothelial dysfunction, and cardiovascular diseases like atherosclerosis, hypertension, and myocardial infarction.

In cancer, gut dysbiosis promotes OS by increasing the generation of pro-inflammatory metabolites, such as secondary bile acids and LPS [61], which eventually leads to the activation of redox-sensitive signaling pathways, such as NF-κB, leading to chronic inflammation and DNA damage [62]. The interplay between gut microbiota and ROS is particularly evident in CRC, where microbial metabolites modulate tumor progression and therapy resistance. OS can negatively impact beneficial gut bacteria, reducing their growth and folate synthesis, which may increase the risk of cancer [63]. Studies have consistently demonstrated significant disruptions in oxidative balance among patients with CRC, marked by decreased antioxidant defenses and elevated OS markers. For instance, reduced levels of total antioxidant capacity (TAC), glutathione (GSH), and critical antioxidant enzymes, such as CAT, are commonly observed in patients with CRC [64]. Concurrently, increased levels of OS markers like malondialdehyde (MDA) and ROS, reflect the heightened oxidative burden in these individuals [65]. Dysbiosis is associated with increased ROS generation and compromised bacterial functions, such as reduced folate synthesis in *Enterococcus durans*, which impacts the effectiveness of cancer treatment [66]. The liver’s exposure to microbiota-associated molecular patterns and bacterial metabolites, such as TMAO and hydrogen sulfide, can disrupt mitochondrial function in liver cells. This dysfunction exacerbates the production of ROS, leading to increased oxidative damage. Transported through the portal vein, these factors further link the gut microbiota to the development of hepatocellular carcinoma [67].

OS and gut microbiota dysbiosis interact in a bidirectional manner, synergizing neurodegenerative processes in Alzheimer’s disease (AD), and Parkinson’s disease (PD). Dysbiosis enhances gut permeability, with bacterial endotoxins like LPS, entering the circulation and triggering systemic inflammation [68]. This initiates microglial activation within the brain via Toll-like receptor 4 (TLR4) and NF-κB signaling, increasing the generation of ROS and pro-inflammatory cytokines, resulting in neuronal damage [69]. In AD, dysbiosis of the gut is related to increased β-amyloid (Aβ) accumulation and tau hyperphosphorylation by OS-mediated mitochondrial dysfunction [70]. Similarly, in PD, the disturbed composition of the gut microbiota disrupts dopamine metabolism and increases α-synuclein aggregation, further accelerating neurodegeneration [71]. In addition, loss of protective microbial metabolites like SCFAs, in particular butyrate, impairs mitochondrial function and antioxidant defenses, which enhance OS [72].

IBD is a multifactorial disorder driven by the interplay of genetic, environmental, microbial, and immunological factors [73]. Among these, dysbiosis and OS are pivotal contributors, each influencing the other to perpetuate inflammation, tissue damage, and disease progression. In IBD, dysbiosis is marked by the depletion of beneficial microbes, such as Firmicutes and Bacteroidetes, and an overrepresentation of pro-inflammatory taxa like Proteobacteria and Enterobacteriaceae [74]. This microbial imbalance disrupts intestinal homeostasis and triggers an inflammatory response. The gut microbiota plays an essential role in maintaining epithelial barrier integrity and modulating immune responses [75]. In dysbiosis, pathogenic bacteria and their metabolites, such as LPS and hydrogen sulfide, compromise tight junctions and weaken the intestinal barrier [76]. This permits microbial translocation and stimulates immune cells, including macrophages and neutrophils, which release pro-inflammatory cytokines and ROS, initiating a vicious cycle of oxidative damage and inflammation [77]. Additionally, dysbiosis disrupts the production of SCFAs, which are essential metabolites formed during the fermentation of dietary fibers. SCFAs, particularly butyrate, are anti-inflammatory and play a role in regulating epithelial cell function and immune tolerance in the gut. Their depletion further exacerbates inflammation and OS [78]. An increase in oxygen levels in the gut has been suggested to contribute to dysbiosis in IBD, shifting the microbiota from obligate anaerobes to facultative anaerobes and disrupting the gut’s normal anaerobic environment [79]. In IBD, OS is a hallmark of mucosal injury and disease activity. Elevated levels of biomarkers such as total oxidant status (TOS) and oxidative stress index (OSI) have been consistently reported in patients with IBD, correlating with disease severity [80]. Another study has reported that high ROS levels also elevate OS markers, such as MDA and 8-hydroxy-2′-deoxyguanosine (8-OHdG), which are correlated with disease severity [81]. ROS activate transcription factors like nuclear factor kappa B (NF-κB) and hypoxia-inducible factor-1 alpha (HIF-1α), driving the production of pro-inflammatory cytokines such as tumor necrosis factor-alpha (TNF-α), interleukin-6 (IL-6), and interleukin-1 beta (IL-1β). These cytokines recruit immune cells to the site of inflammation, creating a feedback loop between OS and inflammation [82]. Meanwhile, excess ROS damages cellular lipids, proteins, and DNA, further compromising the intestinal barrier and promoting epithelial cell apoptosis. This damage exacerbates intestinal permeability, allowing luminal antigens and microbes to penetrate deeper into the intestinal wall, intensifying the immune response and perpetuating inflammation [83]. A study has shown that reduced levels of antioxidants, such as GSH and SOD, in patients with IBD underscore their inability to effectively neutralize ROS [84].

Non-alcoholic fatty liver disease (NAFLD) is a complex condition in which dysbiosis and OS play pivotal roles in its pathogenesis and progression. These factors act synergistically to amplify inflammatory responses and hepatocellular damage [85]. NAFLD-associated dysbiosis is often characterized by an increase in Firmicutes and a decrease in Bacteroidetes, which is correlated with elevated inflammation markers, such as TLR4 [86]. Meanwhile, pathogen-associated molecular patterns (PAMPs), such as LPS from Gram-negative bacteria, can cross the compromised gut barrier and activate hepatic immune responses [87]. Dysbiosis contributes to increased intestinal permeability, or “leaky gut”, allowing microbial metabolites, toxins, and bacterial fragments to enter the bloodstream and reach the liver through the portal vein [88]. This translocation activates hepatic Kupffer cells and hepatocytes, prompting the release of inflammatory cytokines, such as TNF-α and IL-6, which perpetuate chronic inflammation [89]. Additionally, a healthy microbiome produces SCFAs, which play vital roles in regulating lipid metabolism and inflammation [90]. However, in dysbiosis, SCFA production is disrupted, impairing the gut-liver axis and exacerbating metabolic and inflammatory imbalances [91].

Recent advancements have significantly improved our understanding of the intricate relationship between gut microbiota dysbiosis and type 2 diabetes mellitus (T2DM), emphasizing the role of microbiome composition in disease pathogenesis and progression [92]. Research has found correlations between shifts in the gut microbiota and plasma glucose levels, revealing that T2DM is associated with a reduction in beneficial Firmicutes and an overgrowth of potentially pathogenic Proteobacteria [93]. These changes in microbiota composition are thought to contribute to systemic inflammation, insulin resistance, and metabolic dysregulation, all of which are central to the development of T2DM [93]. Furthermore, gut dysbiosis contributes to OS, which is a dynamic factor in the pathogenesis of T2DM. The altered microbial community in the gut promotes the production of harmful metabolites, such as TMAO and LPS, which induce oxidative damage and exacerbate inflammatory responses [94]. These metabolites, together with other byproducts of dysbiosis, activate immune cells that further release pro-inflammatory cytokines and ROS, worsening OS [50]. The imbalance between ROS production and antioxidant protection accelerates tissue damage in insulin-sensitive tissues like the liver and muscle, resulting in insulin resistance [95]. Dysbiosis also facilitates the process by disrupting the gut-liver and gut-brain axes, promoting inflammation and metabolic dysregulation [96]. The gut microbiota plays an important role in the regulation of the immune system and metabolic homeostasis, with alterations in the microbiota driving oxidative stress and inflammatory pathways that drive T2DM development and its complications, including cardiovascular disease, nephropathy, and retinopathy [97].

Cutting-edge research has illustrated the complex relationship between gut microbiota dysbiosis and obesity, highlighting the pivotal role of microbial composition in regulating energy metabolism, inflammation, and appetite [98]. Research has consistently shown that obesity is associated with dysbiosis, including an increased Firmicutes-to-Bacteroidetes ratio, which has been linked to greater energy extraction from food, a factor that contributes to weight gain [99]. Additionally, this altered microbiome is associated with changes in intestinal barrier function and an increased risk of metabolic disorders, including insulin resistance and systemic inflammation [100]. Dysbiosis can impact several metabolic pathways, including the regulation of gut peptides involved in satiety, such as ghrelin and peptide YY. These changes promote a self-sustaining cycle of overeating and metabolic derangement, which accelerates the progression of obesity [101]. The interplay between dysbiosis and energy homeostasis has profound implications for the treatment of obesity. Dysbiotic gut bacteria can alter energy metabolism, contributing to excessive fat deposition and the development of metabolic disorders [102].

Celiac disease (CD) is an autoimmune disorder triggered by the ingestion of gluten by genetically predisposed individuals, particularly those with the HLA-DQ2/DQ8 genotype [103]. This condition is characterized by an inappropriate immune response to gluten, which leads to inflammation and damage to the small intestine [104]. Recent studies have highlighted the important role of intestinal dysbiosis in the pathogenesis of CD, suggesting that the composition and diversity of the gut microbiota can significantly influence the onset and progression of the disease [105]. One of the hallmarks of CD is alteration of the gut microbiota, often referred to as dysbiosis [106]. Beneficial bacteria, such as Bifidobacterium and Lactobacillus, which are known for their anti-inflammatory properties and ability to maintain intestinal barrier function, are found at lower levels in patients with CD [107]. These bacteria are typically involved in modulating the immune system and promoting gut health of the host. Reduced production of SCFAs, especially butyrate, which plays a role in maintaining intestinal integrity, has been noted [108]. Increased levels of harmful microbial metabolites in the blood and urine may contribute to inflammation and tissue damage in the intestine [109]. Furthermore, there is an observed increase in the levels of pro-inflammatory bacteria, such as *Bacteroides*, *Escherichia coli*, and *Staphylococcus*. These bacteria can contribute to inflammation and may influence the immune system’s response to gluten, exacerbating the disease [110]., Eventually elevated ROS levels in patients with CD indicate a state of heightened oxidative activity that contributes to cellular dysfunction [111]. Lipid peroxidation products, such as MDA, are markers of oxidative damage to the cell membrane. Increased levels of these products have been reported in patients with CD, suggesting compromised membrane integrity and cellular health [112]. Oxidative modification of proteins, as evidenced by increased protein carbonylation, reflects broader cellular damage in CD, contributing to the inflammatory processes observed in this disease [111]. In addition to microbial imbalance, patients with CD also exhibit a reduction in IgA-coated bacteria. IgA is an important antibody in mucosal immunity, and reduced IgA coating of bacteria has been linked to impaired immune tolerance and an increased risk of immune-mediated damage [113].

Rheumatoid arthritis (RA), an autoimmune inflammatory disease, has also been increasingly associated with intestinal dysbiosis [114]. This connection is supported by several studies that highlight the critical role of the gut microbiota in modulating immune responses and influencing disease onset and progression [115]. Specific bacterial alterations, such as an increase in *Prevotella copri* (inducing inflammation) and a decrease in *Bacteroides fragilis* (anti-inflammation), disrupt immune homeostasis, fostering disease activity [116]. Gut dysbiosis contributes to impaired intestinal barrier function, systemic immune activation, and mucosal inflammation, exacerbating RA symptoms through autoantibody production and T cell dysregulation [117]. Additionally, the expansion of Th17 cells, influenced by the gut microbiota, drives autoimmune processes [117]. Notably, evidence suggests that dysbiosis may precede RA onset, implicating a potential causal role in its development [114]. Additionally, OS, marked by increased markers like MDA and reduced antioxidants like GSH, synergizes with immune dysregulation to drive RA progression, further linking microbial, oxidative, and immune pathways in the disease process [118]. These findings underscore the potential of integrative approaches targeting the microbiota, OS, and systemic inflammation in RA management.

Kidney function is closely linked to the gut microbiota, which regulates the production of indoxyl sulfate (IS) and indole-3-propionic acid (IPA) through tryptophan metabolism [119]. Under dysbiosis, an increase in proteolytic bacteria accelerates IS generation, which increases in kidney disease and activates AHR and Stat3, creating OS, inflammation, and fibrosis [120]. Alternatively, IPA produced by *Clostridium sporogenes* is an antioxidant that prevents Stat3 phosphorylation and reduces inflammatory and fibrotic gene expressions [121]. Rebalancing dietary or probiotic restoration of gut microbial homeostasis may improve IPA levels but reduce IS burden, offering a therapeutic option to overcome oxidative stress and maintain kidney function [119,120].

**Figure 2:**
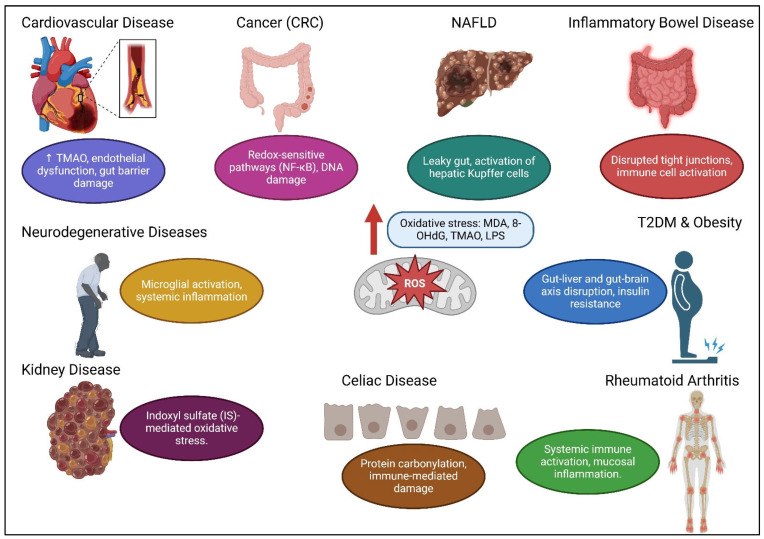
Essential OS mechanisms, their effects on cellular processes, and their roles in the development of various diseases.

Table 2 provides a comprehensive overview of the key biological, metabolic, and immune-related factors involved in the pathophysiology of various diseases, including autoimmune disorders (Crohn’s disease, RA), metabolic disorders (T2DM, NAFLD), neurological disorders (AD, PD), inflammatory disorders (IBD), and cancer (CRC). Figure 2 illustrates the impact of elevated levels of ROS and their association with various human diseases.

## 6. Extracellular Vesicles as Mediators in Signaling Communication

Extracellular vesicles (EVs) are commonly classified as small EVs (sEVs) and large EVs (lEVs) based on their size, biogenesis, and molecular composition (Table 3) [122,123]. sEVs, typically <200 nm in diameter, include exosome-like particles and are largely derived from the endosomal pathway through intraluminal vesicle generation in multivesicular bodies (MVBs) [124]. Conversely, lEVs, often greater than 200 nm, encompass microvesicles (MVs) and apoptotic bodies, which are either budded directly from the plasma membrane or created through cell fragmentation upon apoptosis [125]. While the size distinction between sEVs and lEVs has classically been utilized, it remains based on the separation and characterization techniques employed. Molecular signatures also distinguish these subtypes; sEVs are enriched in tetraspanin (CD9, CD63, and CD81), ESCRT-related proteins (TSG101 and Alix), and lipid raft components, consistent with their endosomal origin [126]. In contrast, lEVs contain markers such as integrins, actinin-4, and ARF6, which are associated with plasma membrane dynamics [127]. Functionally, sEVs are typically involved in intercellular communication, immune modulation, and the transfer of bioactive molecules such as miRNAs, proteins, and lipids [128], whereas lEVs are involved in coagulation, inflammation, and extracellular matrix remodeling [129]. The 2023 Minimal Information for Studies of Extracellular Vesicles (MISEV2023) guidelines highlight the requirement for standardized isolation, characterization, and functional validation to impart reproducibility and biological relevance to EV-related research [123]. With advancing technology, it is essential to decipher the differential roles of sEVs and lEVs and enhance the classification criteria to unlock their potential for therapeutics and diagnostics [130,131,132].

### Interaction of EVs in Gut Microbiota and OS

EVs bearing cytolethal distending toxin (CDT) from *Campylobacter jejuni* modulate the progression of CRC, although their action may be context-specific. Caco-2 cells in CRC were inhibited for proliferation by EVs loaded with CDT, with induction of mid-level apoptosis and autophagic reactions, suggesting its therapeutic potential for cancer. However, CDT, through chronic exposure in the context of gut microbiota dysbiosis, can contribute to CRC progression by inducing DNA damage, promoting inflammation, and facilitating immune evasion. Its effects may vary depending on the context of the disease and duration of exposure [133].

In addition, microbial-derived EVs (MDEVs) play a crucial role in maintaining gut health by modulating gut microbiota composition and reducing OS, an important factor associated with various GIT and systemic diseases [134]. These vesicles serve as vital communicators between microorganisms in the gut and host cells, carrying bioactive molecules, such as proteins, lipids, RNA, and metabolites [135]. By influencing microbial interactions, immune responses, and intestinal barrier integrity, MDEVs are essential for maintaining gut homeostasis and preventing inflammation-related disorders [136].

One of the prominent functions of MDEVs is their involvement in reducing OS in the gut, which is essential for maintaining the health of the intestinal mucosa [135]. MDEVs have shown antioxidant effects by transferring specific molecules like antioxidants, regulatory proteins, and non-coding RNAs, to host cells, thereby enhancing cellular defenses against oxidative insults [136]. Certain strains of Lactobacillus and Bifidobacterium produce EVs that contain enzymes like SOD, CAT, and GPx. These enzymes directly scavenge ROS, helping to reduce OS [137]. By transferring antioxidant enzymes to intestinal epithelial cells (IECs), MDEVs protect these cells from oxidative damage. Additionally, small non-coding RNAs, such as microRNA (miR-199a-3p), are also transported in MDEVs, which regulate OS pathways at the post-transcriptional level, downregulating pro-inflammatory mediators and promoting the repair of damaged cells in the gut [138].

MDEVs also carry metabolites like SCFAs, which are produced during fiber fermentation by the gut microbiota. SCFAs enhance antioxidant defense and restore intestinal barrier integrity, preventing inflammation and oxidative damage. These metabolites also promote the expression of tight junction proteins (TJPs), such as zona occludens 1 (ZO-1) and occludin, which are crucial for maintaining intestinal permeability and preventing the leakage of inflammatory molecules into the bloodstream [139,140].

MDEVs regulate the gut immune response to OS through interactions with immune cells, including macrophages, dendritic cells (DCs), and T cells. For example, EVs derived from Lactobacillus, like *Lactobacillus johnsonii*, *Lactobacillus plantarum*, and *Lactobacillus reuteri*, have been shown to inhibit pro-inflammatory cytokines, induce the Nrf2/HO-1 pathway, and restore gut homeostasis [141]. These immune-modulating activities are particularly significant in conditions like colitis, where OS is implicated in chronic gut inflammation [142]. Additionally, *Bacteroides thetaiotaomicron*-derived EVs modulate immune cells via TLR pathways with varying consequences in healthy and IBD conditions [143]. Overall, MDEVs play a role in stabilizing the gut microbiota by inducing beneficial bacteria and inhibiting pathogenic species, thereby promoting a healthier gut ecosystem and alleviating IBD symptoms [141,142].

In the context of neurological diseases, OS-modified EVs mediate gut-brain interactions, exacerbating neuroinflammation in disorders like AD and PD. Gut microbiota-derived EVs enriched in LPS stimulate TLR4 signaling in microglia and astrocytes, thereby promoting neuroinflammation [144]. Moreover, SCFA-deficient EVs, due to dysbiosis, reduce neuroprotective signaling, further impairing gut-brain homeostasis [72]. Additionally, microbiota-derived EVs can carry neurotoxic metabolites, including amyloid-like proteins, which may accelerate protein aggregation in neurological diseases [145]. OS-enhanced EV uptake across the blood-brain barrier (BBB) allows gut-derived inflammatory and neurotoxic molecules to directly impact neurons and glial cells, sustaining microglial activation and systemic mitochondrial dysfunction [146].

MDEVs also facilitate gut-brain communication, an essential pathway for maintaining immune and neurological balance [147]. For example, *Bacteroides fragilis*-derived EVs contain polysaccharide A, which modulates the host immune response and regulates inflammation in the gut [148,149]. This interaction may influence neuroinflammatory conditions, such as multiple sclerosis [149]. Moreover, EVs from *Akkermansia muciniphila* enhance gut barrier integrity by increasing the expression of TJPs, thus preventing harmful bacteria and toxins from entering the circulation. This helps reduce systemic inflammation, which may contribute to neurological disorders like anxiety and depression [150].

However, EVs can also play a role in promoting OS in certain contexts. EVs transmit OS through multiple pathways, depending on their cell of origin and composition. sEVs, which are produced via the endosomal pathway, are ceramide-rich, a bioactive lipid that transmits mitochondrial dysfunction and apoptotic signals through interactions with sphingomyelinases and induction of OS-mediated cell death [151]. Mitochondrial-derived vesicles (MDVs), secreted from stressed mitochondria following excessive production of ROS, contain oxidized mitochondrial constituents, including lipids, mitochondrial DNA (mtDNA), and misfolded proteins, which increase oxidative stress in target cells by inducing ROS generation, mitochondrial homeostasis disturbance, and apoptosis, as observed in diabetic foot ulcers [152]. EVs released from senescent or stressed red blood cells, specifically in T2DM and cardiovascular diseases, cause endothelial dysfunction by delivering heme, free iron, and oxidative enzymes that catalyze ROS generation, disrupt NO signaling, and upset endothelial homeostasis [153]. Platelet-derived EVs (pEVs), generated from activated platelets under pro-inflammatory and OS conditions, convey procoagulant and pro-inflammatory mediators, such as thromboxane and NADPH oxidase subunits, which enhance oxidative stress, cause endothelial cell dysfunction by increasing ROS production, and reduce antioxidant defenses [132,154]. Tumor-derived EVs (TDEVs), secreted by cancer cells in their microenvironmental niches, carry oncogenic proteins, oxidative stressors like Nrf2 inhibitors, and miRNAs that repress antioxidant pathways in target cells to induce tumor growth by promoting oxidative DNA damage and evading immune cells [155]. These EVs communicate oxidative stress through various mechanisms, including ceramide signaling, where ceramide-rich sEVs activate sphingomyelinases, destabilizing mitochondrial integrity and triggering apoptosis [151]. During mitochondrial dysfunction, MDVs deliver dysfunctional mitochondrial components to target cells, triggering ROS accumulation and metabolic stress [156]. When endothelial dysfunction occurs, erythrocyte-derived EVs suppress endothelial NO signaling and increase vascular oxidative stress, which in turn triggers endothelial cell apoptosis, inflammation, and procoagulant activity [153]. At the same time, pEVs induce oxidative damage in vascular and immune cells, leading to thrombotic and inflammatory disorders [154].

Despite these mixed findings, research on MDEVs is still in its early stages, and further exploration is needed to understand the precise mechanisms by which these vesicles mediate OS responses in the gut. The therapeutic potential of MDEVs in managing OS-related diseases such as IBD, CRC, and metabolic disorders remains largely unexplored [157]. More in-depth studies are needed to identify the specific components of MDEVs that modulate OS and their interactions with host cells.

**Figure 3:**
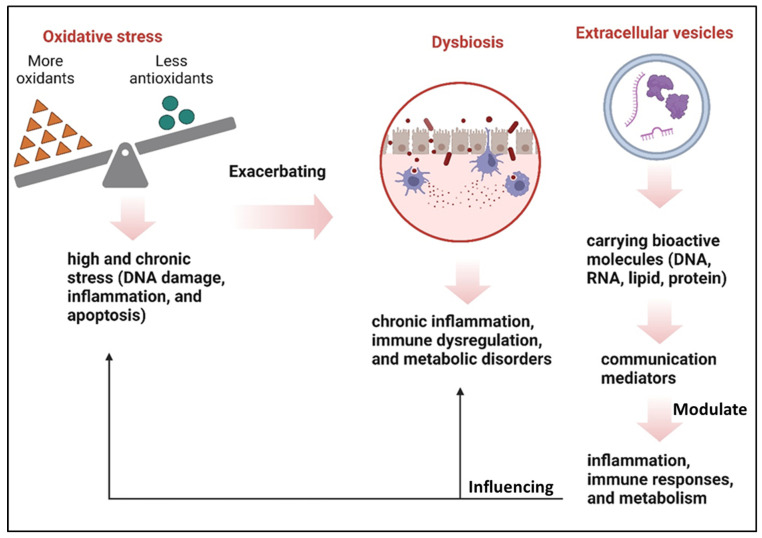
The relationships between oxidative stress, gut microbiota, and EVs and their roles in disease progression.

Figure 3 illustrates the complex interplay between OS, gut microbiota, and EVs in various diseases. OS, caused by an imbalance between ROS and antioxidants, leads to cellular damage and disease development. Dysbiosis of the gut microbiota, influenced by OS, contributes to inflammation and metabolic dysfunction. EVs act as integral components of molecules between the gut and host tissues and modulate immune responses and metabolism. Table 4 summarizes the roles of different EVs in modulating OS within the gut microbiota and beyond. It categorizes EVs based on their sources, effects on OS ( protective or harmful), mechanisms of action, and associations with various diseases. Notably, MDEVs from probiotics such as Lactobacillus and Bifidobacterium, help mitigate OS through antioxidant enzyme transfer and immune modulation, whereas EVs from cancer cells, mitochondria, red blood cells, and platelets contribute to OS by transferring oxidative molecules, impairing endothelial function, and inducing inflammatory responses.

## 7. Personalized Therapeutic

Individualized therapy protocols, such as probiotics, antioxidants, and fecal microbiota transplantation (FMT), are effective interventions against OS and gut microbiota-related diseases [158]. Prebiotics and probiotics support the restoration of the gut barrier, control inflammation, and neutralize OS by modulating the generation of ROS, maintaning antioxidant defenses, and maintaning the intestinal barrier [159,160]. Targeting Lactobacillus and Bifidobacterium strains using probiotics or FMT has been promising in preventing age-associated inflammation and oxidative stress [161]. Probiotics also regulate ROS-mediated diseases such as IBD and CRC [162]. Combined as synbiotics, prebiotics enhance the survival of probiotics, creating synergistic gut health [163,164].

Similarly, antioxidants shield cells against OS by scavenging free radicals and inhibiting cellular damage associated with cancer, cardiovascular disease, and neurodegenerative diseases [165]. Dietary polyphenols, vitamins C and E, and antioxidant phytochemicals can complement microbiota-directed therapies to reconstitute gut health, mitigate OS, and enhance the intestinal barrier [166]. Notably, gut microbiota enhance host antioxidant defenses by producing reactive sulfur species, such as hydrogen sulfide, whose concentration can be supplemented with cystine to prevent OS and liver damage [167,168].

While FMT is a promising treatment for gut dysbiosis, biosafety concerns limit its therapeutic application [169]. Recent findings suggest that gut microbiota-derived extracellular vesicles (Gm-EVs) may be a potential alternative. In a mouse model of ulcerative colitis (UC), Gm-EVs originating from conditioned microbiota, which were tea leaf lipid/pluronic F127-coated curcumin nanocrystals (CN@Lp127s), showed superior efficacy compared to FMT in reducing colonic inflammation, restoring barrier function, rebalancing gut microbiota, and regulating purine metabolism, leading to decreased uric acid levels and improved UC outcomes [170,171]. These findings highlight the potential of nanomedicine-trained Gm-EVs as a safer and more effective alternative to FMT for UC treatment.

Future precision medicine strategies, in line with microbiome profiling, OS markers, and genetic risk factors, aim to provide maximum treatment with minimal risk. Engineered EVs for the targeted delivery of drugs and optimized FMT substitutes like Gm-EVs, can revolutionize the therapy of OS-related and gut microbiota-associated diseases into new territories in personalized health.

## 8. Conclusions

In conclusion, the bidirectional relationship between OS and the gut microbiota plays a pivotal role in the pathogenesis of various diseases, including metabolic, inflammatory, and neurodegenerative disorders. Excessive production of ROS induces gut dysbiosis, which in turn exacerbates OS, creating a vicious cycle that accelerates disease progression. Conversely, maintaining balanced ROS levels supports gut homeostasis, enhances immune function, and prevents the overgrowth of pathogenic microorganisms. The emerging role of EVs, particularly those derived from microbes, as mediators of OS and gut microbiota communication, highlights their potential as novel therapeutic targets. EVs act as bioactive carriers, facilitating intercellular communication and regulating redox balance. Depending on the context, they can either protect against or amplify oxidative damage in different disease states. Understanding the mechanisms by which EVs mediate the interaction between OS and the gut microbiota opens new avenues for therapeutic strategies. Approaches such as probiotics, prebiotics, antioxidants, and microbiota-targeted therapies may help restore microbial balance and mitigate OS. Future research should aim to uncover the precise roles of MDEVs in maintaining gut health and modulating OS, with potential clinical applications in managing OS-related diseases. By targeting these intricate interactions, it may be possible to develop innovative strategies to alleviate the disease burden and enhance overall health.

## Figures and Tables

**Table 1:** Gut microbiota changes in various health conditions. ↑ indicates an increase, while ↓ indicates a decrease in gut microbiota changes.

Health Condition	Gut Microbiota Changes	Potential Effects	References
**Irritable Bowel Syndrome (IBS)**	↑ *Enterobacteriaceae*, *Lactobacillaceae*, *Bacteroides* ↓ *Faecalibacterium*, *Bifidobacterium*	Abdominal pain, bloating, altered bowel habits	[36,37]
**Pancreatic Diseases**	↑ *Escherichia-Shigella, Firmicutes and Actinobacteria* ↓ *Faecalibacterium*, *Bifidobacterium*	Chronic inflammation, pancreatic fibrosis, increased cancer risk	[38]
**Obesity**	↑ *Bacillota/Bacteroidetes* ratio (>1) ↓ *Faecalibacterium prausnitzii*	Increased obesity risk, insulin resistance	[39]
**Anxiety Disorders**	↓ Microbial richness and diversity ↓ *Bacillota* ↑ *Bacteroidetes*, *Fusobacteria*	Associated with higher anxiety levels	[40]
**Depression**	↓ *Dialister*, *Coprococcus* ↑ *Klebsiella*	Linked to depressive symptoms	[41]
**Bipolar Disorder**	*↓ Faecalibacterium, Ruminococcaceae, Christensenellaceae* ↑ *Clostridiaceae*, *Collinsella, Flavonifractor, Pseudomonadaceae*	Associated with bipolar disorder symptoms	[42]
**Autism Spectrum Disorder (ASD)**	↑ *Clostridium* species	May influence ASD symptoms	[43]
**Schizophrenia**	↑ *Proteobacteria* ↓ *Faecalibacterium*	Associated with schizophrenia symptoms	[44]
**Atherosclerosis & Coronary Artery Disease**	↑ TMAO-producing bacteria, *Enterobacteriaceae*	Promotes systemic inflammation, endothelial dysfunction, lipid imbalance	[45]
**Heart Failure (HF)**	↓ *Faecalibacterium prausnitzii*, SCFA producers ↑ Gram-negative bacteria (*Escherichia*, *Shigella*)	Increased intestinal permeability, systemic inflammation, worsening cardiac function	[46]
	↑ *Blautia*, *Dialister* (in milder cases) ↓ *Bacteroidota*, ↑ *Pseudomonadota* (in older patients)		[47]
	Changes in *Ruminococcus*, *Collinsella*, *Eubacterium*, *Lachnospiraceae*		[48]

**Table 2:** The interconnectedness of factors and their role in driving chronic inflammation, metabolic disturbances, microbial imbalances, and immune dysregulation in various diseases.

Disease	Microbiota Changes	Oxidative Stress Markers	Key Interactions	References
**Cardiovascular Disease**	• Loss of microbial diversity • Increase in pro-inflammatory species • Decreased SCFA production	• Increased ROS • Elevated TMAO • Increased LPS	• Compromised gut barrier allows bacterial toxins to enter circulation • Reduced SCFAs	[54,55,56,57,58,59,60]
**Cancer (particularly CRC)**	• Increased pro-inflammatory metabolites • Elevated secondary bile acids and LPS	• Decreased TAC, GSH, and CAT • Increased MDA and ROS	• Activation of redox-sensitive pathways (NF-κB) • Chronic inflammation and DNA damage	[61,62,63,64,65,66,67]
**Neurodegenerative Diseases (AD, PD)**	• Enhanced gut permeability • Disturbed gut microbiota composition	• Increased ROS generation • OS-mediated mitochondrial dysfunction	• Bacterial endotoxins trigger systemic inflammation • Microglial activation via TLR4 and NF-κB	[68,69,70,71,72]
**Inflammatory Bowel Disease (IBD)**	• Depletion of beneficial microbes (Firmicutes, Bacteroidetes) • Overrepresentation of pro-inflammatory taxa (Proteobacteria, Enterobacteriaceae)	• Elevated TOS and OSI • Increased MDA and 8-OHdG • Reduced GSH and SOD	• Compromised tight junctions and intestinal barrier • Microbial translocation stimulates immune cells	[73,74,75,76,77,78,79,80,81,82,83,84]
**Non-alcoholic Fatty Liver Disease (NAFLD)**	• Increased Firmicutes • Decreased Bacteroidetes • Increased intestinal permeability	• Elevated TLR4 • Increased PAMPs (e.g., LPS)	• Microbial metabolites enter bloodstream through “leaky gut” • Activation of hepatic Kupffer cells and hepatocytes	[85,86,87,88,89,90,91]
**Type 2 Diabetes Mellitus (T2DM)**	• Reduced beneficial Firmicutes • Overgrowth of pathogenic Proteobacteria	• Increased ROS	• Systemic inflammation and insulin resistance • Disruption of gut-liver and gut-brain axes	[92,93,94,95,96,97]
**Obesity**	• Increased Firmicutes-to-Bacteroidetes ratio • Altered intestinal barrier function	• Systemic inflammation	• Greater energy extraction from food • Changes in gut peptides involved in satiety (ghrelin, peptide YY)	[98,99,100,101,102]
**Celiac Disease (CD)**	• Lower levels of beneficial bacteria (Bifidobacterium, Lactobacillus) • Increased pro-inflammatory bacteria (Bacteroides, E. coli, Staphylococcus)	• Elevated ROS levels • Increased lipid peroxidation products (MDA)	• Oxidative modification of proteins (protein carbonylation) • Immune-mediated damage	[103,104,105,106,107,108,109,110,111,112,113]
**Rheumatoid Arthritis (RA)**	• Increased Prevotella copri (pro-inflammatory) • Decreased Bacteroides fragilis (anti-inflammatory)	• Increased MDA	• Disrupted immune homeostasis • Systemic immune activation • Mucosal inflammation	[114,115,116,117,118]
**Kidney Disease**	• Increased proteolytic bacteria • Altered tryptophan metabolism	• Increased indoxyl sulfate (IS)	• IS activates AHR and Stat3 pathways • IPA (antioxidant) prevents Stat3 phosphorylation	[119,120,121]

**Table 3:** The latest classification of extracellular vesicles.

Feature	Small EVs (sEVs)	Large EVs (lEVs)
Size	<200 nm	>200 nm (typically 200–1000 nm, including apoptotic bodies >1000 nm)
Biogenesis	Endosomal origin (formed via multivesicular bodies—MVBs and released by exocytosis)	Plasma membrane shedding or direct budding; apoptotic bodies result from cell fragmentation
Isolation Methods	Ultracentrifugation, size-exclusion chromatography (SEC), density gradient centrifugation	Differential centrifugation, filtration, size-exclusion chromatography
Membrane Markers	CD9, CD63, CD81 (tetraspanins)	Integrins (ITGB1, ITGA2B), Annexins (ANXA5), Flotillin-1 (FLOT1)
Cytoskeletal Proteins	Actin, TSG101, ALIX	Actinin-4 (ACTN4), Tubulin, Myosin
Lipid Raft-Associated Proteins	Flotillin-1 (FLOT1), Caveolin-1 (CAV1)	Flotillin-1 (FLOT1), Caveolin-1 (CAV1) (also found in lEVs)
Membrane Trafficking Proteins	Rab GTPases (Rab27a, Rab5), ESCRT components (TSG101, ALIX)	ARF6, VAMP3, Rab22A
Apoptotic Markers	Absent (unless from dying cells)	Histones (H3, H2B), Caspase-3, Annexin V (specific to apoptotic bodies)
Mitochondrial Markers	Usually absent or very low	TOMM20, ATP5A (may be enriched in lEVs, but should be validated to avoid contamination)
RNA Content	Enriched in miRNAs, lncRNAs, mRNAs	Contains mRNAs, rRNAs, and some miRNAs but varies based on origin
Protein Content	Enriched in cytosolic and membrane proteins (e.g., ALIX, TSG101, CD63)	Contains cytoskeletal and apoptotic proteins (e.g., actin, caspases, histones)
Functional Roles	Intercellular communication, cargo delivery, immune modulation, tumor progression	Cell signaling, immune modulation, removal of cellular debris, apoptosis

**Table 4:** Different types of EVs involved in gut microbiota-OS interactions.

Type of EV	Source	Effects on OS	Mechanisms	Associated Diseases
MDEVs	Lactobacillus, Bifidobacterium	Reduce OS	Antioxidant enzyme transfer (SOD, CAT, GPx), SCFAs, miRNA-mediated OS regulation	IBD, CRC, metabolic disorders
sEVs	Cancer cells, immune cells	Promote OS	Ceramide signaling, oxidative DNA damage, immune evasion	Cancer progression
MDVs	Stressed mitochondria	Promote OS	Transfer of oxidized mitochondrial constituents, mitochondrial dysfunction	Diabetic foot ulcers, metabolic diseases
Erythrocyte-derived EVs	Senescent RBCs	Promote OS	Free iron, heme, oxidative enzymes disrupt NO signaling	CVD, Type 2 diabetes
Platelet-derived EVs	Activated platelets	Promote OS	Thromboxane, NADPH oxidase activation, vascular oxidative stress	Thrombotic disorders, inflammation

## Data Availability

There is no data to support the findings of this review.
